# Reply to: Reinterpretation of purported molting evidence in the Thermopolis *Archaeopteryx*

**DOI:** 10.1038/s42003-021-02367-9

**Published:** 2021-07-05

**Authors:** Thomas G. Kaye, Michael Pittman

**Affiliations:** 1 Foundation for Scientific Advancement, Sierra Vista, AZ USA; 2 Vertebrate Palaeontology Laboratory, Research Division for Earth and Planetary Science, The University of Hong Kong, Pokfulam, Hong Kong SAR, China

**Keywords:** Evolution, Palaeontology

**Replying to**: Y. Kiat et al. *Communications Biology* 10.1038/s42003-021-02349-x (2021)

Kiat et al. have presented a response to our analysis of feather sheaths in *Archaeopteryx*. They present two major counter arguments in their response: a, that the observed sheaths are simply typical calami, and b, that the observed molting pattern is random. These two arguments are diametrically opposed, if one is true the other cannot be and vice versa. Regardless, we will answer each one in turn. We both agree that the sheaths are part of the animal and not foreign objects. We also agree (based on argument b) that what is represented is molting, it is the molt strategy that is in debate.

Kiat et al. claim that the observed sheaths are simply calami normally covered by the coverts. Analysis of the phosphorus elemental map of WDC-CSG-100^1^ shows that the sheaths in question have very high levels of phosphorus. Based on the brightness of the elemental map in Fig. 1A of^1^, the phosphorus levels in the sheaths have a closer brightness level to that of the bone than with the rachis traces further away. The sheaths also exhibit clearly defined edges indicating a high level of phosphorus saturation. Phosphorus is known to be highly fluorescent and would be the prime candidate for the fluorescence in the images.

**Fig. 1:**
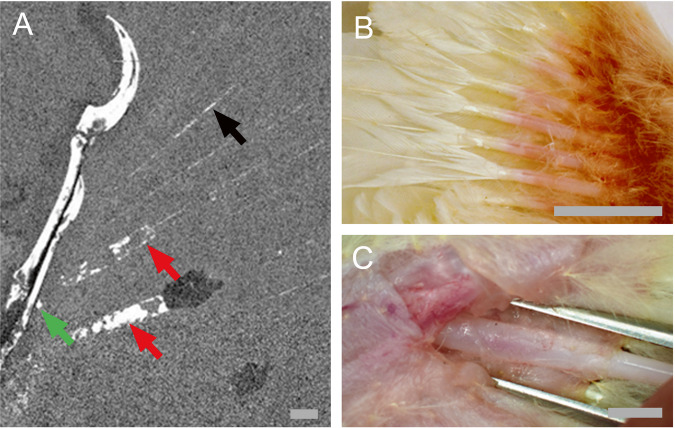
Overview of feather sheaths on baby chick and phosphorus map of WDC-CSG-100. **A** Phosphorus (P) map from Bergmann et al. ^1^ showing high concentration of P in the sheaths vs trace lines of the extended rachis. The two sheaths (red arrows) are also shown separated by one feather. Black arrow is pointing to a feather rachis. Green arrow points to sheath remnant extending to the bone. **B** Chick feather sheaths in ventral view showing growing feathers and sheath extensions outside of patagia. **C** Dissected chick patagia showing the feather sheath continuing through the patagia up to the wing bone. Scale bars are 5 mm.

Kiat et al. also claim that “there is no reason why the sheath would be more likely to preserve than the much thicker calamus”. What is being measured is the residual elemental signature remaining in the matrix. This is unlike the bones which are in fact preserved in three dimensions. The images of the sheaths are based on the residual elemental fingerprint of the original feather complex.

The extended feathers however, just show fine traces of phosphorus from the calami extending the length of the rachis without clearly defined edges (black arrow in Fig. 1A). For the sheaths in question to be remnants of calami, it would require that all the other feathers originally contained an equivalent high level of phosphorus. All the other feathers would need to lose phosphorus due to taphonomic and/or diagenetic processes with only the three remaining sheaths in question retaining the higher original amount. It would also require that the skeleton was subjected to the same depletive processes as the feathers. However, the skeleton itself shows no indications of phosphorus depletion. This scenario requires a transport mechanism that would remove most of the phosphorus and still leave faint traces along the length of the feather rachis. As the other calami still show faint traces of phosphorus leading to the bone, this scenario requires that the sheath structures in question would have been cleared out, but the centerline phosphorus was spared. However, a highly localized process of phosphorus preservation would not explain the preservation on both sides of the body. The sheaths stop abruptly along the length of the feathers and all are of similar length and in similar positions relative to the wing, something not expected in a random preservation process.

Kiat et al. make the point that the sheath and the calami are made from the same keratinous material so one is just as likely to preserve as the other. However, they do not consider the proportion of phosphorus in the two different elements - sheath vs rachis/calami - that would remain after the breakdown of the organics during the preservation process. In an elemental study of modern feathers across multiple specimens, the phosphorus content was found to be in the region of 905 -1357 ppm^2^. In the Bergmann et al.^1^ study of the Thermopolis *Archaeopteryx* specimen, the feather tip showed 1732 ppm and the base 2706 ppm^3^. These numbers are in keeping with the faint rachis lines containing the original level of P. An elemental analysis of the chick sheaths and calamus was done under the electron microscope using energy dispersive spectroscopy (EDS) [EDS data in Supplementary Information]. The quantification results in weight% were ratioed against sulfur and show that the sheath contained proportionally ~5 times more phosphorus than the rachis. This suggests that the faint phosphorus traces match modern levels and sheaths would leave a larger phosphorus fingerprint than the rest of the feather.

The literature shows that the elemental phosphorus in the early-diverging avialan *Anchiornis* is nearly undetectable even though the feathers are better preserved than in *Archaeopteryx*^4^. An elemental map of the troodontid *Jianianhualong* only shows phosphorus on the skeleton and not on the feathers^5^. Of the hundreds of feathered theropods examined under intense Laser-Stimulated Fluorescence (LSF)^6–9^, none showed any feather fluorescence approaching the reaction of the bone.

Kiat et al. indicates that the sheaths coming so close to the bone is problematic. They also claim that the sheath structures would “include portions both inside and outside the postpatagium” and would thus represent calami. A chick with growing feathers and sheaths was dissected to answer this question. Figure 1C shows that the chick’s feather sheaths continue into the postpatagium up to the wing bone and continues outside the postpatagium as viewed in Fig. 1B, demonstrating that the sheaths do exist interior and exterior to the patagium.

Kiat et al. and Kaye et al. both describe multiple molting strategies, sequential and center out as well as Kiat et al.’s description of “no predictable sequence or direction” which we identify in our original paper as “random” molting. We agree that the complete molting sequence in paravians is unknown. In these circumstances the most parsimonious scientific answer is the one that is statistically most likely based on the available data. As an example, cladistics which is widely used in paleontology, gets its answer from the most statistically probable outcome. To test which molting pattern is most statistically likely in this case, we ran a Monti Carlo simulation of random molting across the 11 feathers of both wings (description of mathematical process in Supplementary Information). The molt pattern described with the feather sheaths of WDC-CSG-100 appeared randomly 138 times out of 901,780 random trials showing molting feathers. The probability of finding the WDC-CSG-100 molting pattern in a fossil by chance, equals 1 out of 6534. For a numerically sequential molt the statistics are 0 out of 11. In a center-out molting strategy one would see matched missing feathers in the following positions: 5 and 7, 4 and 8, 3 and 9, 2 and 10, 1 and 11. In this sequence there is a ~1 in 5 chance of detecting a center out molt. Statistically the center-out strategy is the most likely in WDC-CSG-100 based on evidence of feather sheaths in positions 5 and 7.

The primary feather count of the Thermopolis *Archaeopteryx* specimen was at least 11 following Figure 3 of Mayr et al.^10^, which is close to the highest known count of 12 recorded in the 11th *Archaeopteryx* specimen^11^. The data in Kaye et al. therefore shows the sheaths separated by one feather which is not contradicted in Kiat et al.’s argument and they are near the center of the primary series, which still statistically supports the center-out molting argument.

In this rebuttal we provide additional information to reiterate that elements we identify in the Thermopolis *Archaeopteryx* are feather sheaths which have a nature that suggests the use of a center-out molting strategy by this early bird. We do so by showing that the phosphorus levels in the specimen favor sheaths, that their morphology is consistent with modern sheaths and when tested statistically, that the center-out molting strategy is the most parsimonious strategy to explain the data.

## Reporting summary

Further information on research design is available in the Nature Research Reporting Summary linked to this article.

## Supplementary information

Supplementary Information

Reporting Summary

## Data Availability

IDL code available on request from the authors.
