# Supplementary material for: Reply to: Reinterpretation of purported molting evidence in the Thermopolis Archaeopteryx
Source: Commun Biol. 2021 Jul 5;4:839. doi: 10.1038/s42003-021-02367-9 (PMC8257677; doi:10.1038/s42003-021-02367-9)
Supplement: Supplementary file 1 — Supplementary Information [file 42003_2021_2367_MOESM1_ESM.pdf]

Supplementary Information

Supplementary Methods

Monti Carlo simulation

The Monti Carlo simulation was designed as follows. The software program used was Interactive Data Language (IDL) from L3Harris Geospatial. The IDL RANDOMU function uses the Mersenne Twister (MT19937) algorithm[1] (see http://www.math.sci.hiroshima-u.ac.jp/~m-mat/MT/emt.html for details) which according to L3Harris is optimized for Monti Carlo simulations[2]. The program utilized the sub program RandomNumberGenerator_define.pro written by Fanning Software Consulting. In practice no software based pseudo-random number generators are perfectly random because they depend on an algorithm but the Mersenne Twister algorithm has a period of 2^{19937} which far exceeds the number of trails for this simulation and is popular in Monti Carlo simulations[3].

For each trial, two random number sequences eleven digits long (one digit for each feather) were generated, one number for each wing. Then for each trial generation, the numbers were scanned for zeros which represented a random “molting feather”. If either of the numbers had a zero, then that trial was added to the total pool of random molt sequences. The pool of random molts was then examined for trials that matched the specific pattern of the feather sheaths on WDC-CSG-100.

Two programs were used in this simulation, each generating the random sequences in identical ways and starting with the same seed values. One program scanned for zeros anywhere in the numbers, the other scanned for the molt sequence in WDC-CSG-100 so one produced the total pool count and the other produced the number of matches to the fossil molt sequence. One million trials were run which produced a molting pool count of 901,780 out of 1,000,000. The matches to WDC-CSG-100 produced 138 out of 901,780. Software programs available on request.

Energy dispersive x-ray spectroscopy

Energy dispersive x-ray spectroscopy (EDS) of the feather sheath and rachis was done with a JEOL T300 electron microscope and an EDAX silicon drift detector. Samples were taken at 15kV and processed through EDAX Team software for peak identification, background subtraction and ZAF quantification. Quantification results based only on sulfur and phosphorus shown in Supplementary Table 1.

Supplementary Table 1: EDS ratio of phosphorus to sulfur.

Weight%	**Chick Sheath**	**Chick Calamus**
Sulfur	85.62	96.89
Phosphorus	13.48	3.11
Ratio	6.35/1.0	31.15/1.0

Cadaveric dissection

A baby chick cadaver was dissected following standard practices. The chick was purchased from CAL Ranch in Sierra Vista, Arizona and had died of natural causes.

Supplementary References

1 Matsumoto, M. & Nishimura, T. Mersenne Twister: A 623-dimensionally equidistributed uniform pseudorandom number generator. ***ACM Transactions on Modeling and Computer Simulation*** **8**, 3-30 (1998).

2 Harris-Geospatial-Solutions. ***RANDOMU***, <https://www.l3harrisgeospatial.com/docs/randomu.html> (2020).

3 Harrison, R. L. Introduction to Monte Carlo Simulation. ***AIP Conference Proceedings*** **1204**, 17-21 (2010).
